# Dietary habits, nutritional knowledge, and nutritional status among cardiological patients, including those with obesity and diabetes

**DOI:** 10.3389/fnut.2024.1455236

**Published:** 2024-10-02

**Authors:** Anna-Maria Sapała, Wiktoria Staśkiewicz-Bartecka, Elżbieta Grochowska-Niedworok, Marek Kardas

**Affiliations:** ^1^Department of Dietetics and Food Science, Faculty of Science, Natural and Technical Sciences, Jan Długosz University in Częstochowa, Czestochowa, Poland; ^2^Department of Food Technology and Quality Evaluation, Department of Dietetics, Faculty of Health Sciences in Bytom, Medical University of Silesia in Katowice, Zabrze, Poland; ^3^Department of Health Sciences and Physical Culture, University of Applied Sciences in Nysa, Nysa, Poland

**Keywords:** dietary habits, nutritional knowledge, nutritional status, cardiological patients, obesity, diabetes

## Abstract

**Background:**

Cardiovascular disease is a leading cause of death worldwide. The increase in patients with obesity and diabetes raises the risk of cardiovascular diseases. Proper eating habits and adequate nutritional knowledge play a key role in preventing and treating these conditions. This study aimed to evaluate the dietary habits, nutritional knowledge, and nutritional status of patients hospitalized in a cardiology department in Poland, including those with obesity or diabetes.

**Methods:**

The study was conducted at St. Barbara Regional Specialized Hospital No. 5 in Sosnowiec from January to June 2021, involving 301 patients, 154 women (51.2%) and 147 men (48.8%), aged 29 to 87. Participants were assessed for BMI, NRS 2002 scale, morphology, biochemistry results, blood pressure, and examined for nutritional knowledge and habits using proprietary questionnaires. A proprietary scale was used to assess eating habits.

**Results:**

Most cardiology patients were overweight or obese, with 80% exceeding the normal weight range. No significant gender differences were noted in malnutrition risk on the NRS 2002 scale. The study found patients rarely consumed recommended amounts of vegetables, fruits, legumes, whole grains, fish, and dairy products. Only 26.2% regularly ate a second breakfast, and just 9.3% chose water with meals. However, consumption of salty snacks, energy drinks, and alcohol was low. Biochemical and blood test analysis did not show significant differences between patients with diabetes, obesity, and others.

**Conclusion:**

Most cardiology patients were overweight or obese, which poses a significant risk for further health complications, including cardiovascular diseases. Although patients with diabetes and/or obesity had better nutritional knowledge in some areas, this did not lead to healthier eating habits. The absence of significant differences in biochemical tests suggests that overall lifestyle and diet are crucial to cardiovascular health.

## 1 Introduction

The development of civilization and scientific and technological advances are resulting in an increase in the health consciousness of society and an improvement in the living conditions of many populations. At the same time, this development is accompanied by the development of many diseases, of which metabolic diseases are an increasing problem (1, 2). Obesity is a chronic metabolic disease characterized by increased body weight due to a significant increase in body fat. Its basis may be genetic, endocrine, and environmental factors, however, it is most often the result of an excessive energy supply with food (2, 3). Obesity is considered an epidemic, causing dysfunction and pathological conditions of organs and entire systems in the human body over time. These dysfunctions, if not taken care of by specialists, can lead to permanent disability and even indirectly to death (4). This is common, especially in highly developed countries in which the percentage of obese people is higher due to easier access to high-calorie products with high amounts of simple sugars and fat. However, it should be noted that obesity has become a pathogenic factor in the development of type 2 diabetes, hypertension, or depression also in low- and middle-income countries (4, 5). Abdominal obesity, often referred to as central obesity, is characterized by fat accumulation in the abdominal and trunk area. It is particularly dangerous because of its various associated health risks (5). Abdominal obesity is assessed indirectly by anthropometric measurements and indices and directly by MRI, body composition analysis, or CT scans. Methods for assessing abdominal obesity, along with criteria for diagnosis, are as follows: WHR (Waist Hip Ratio—an indicator of body fat distribution) ≥ 0.85 in women, ≥ 1.0 in men; WHtR (Waist to Height Ratio—waist-to-height ratio) ≥ 0.50; abdominal sagittal dimension ≥25,2 cm in women, ≥ 22,8 cm in men; waist circumference ≥ 80 cm in women, ≥ 94 cm in men and direct methods—tissue cross-sectional area at the level of the L4-L5 intervertebral space (6, 7).

In Europe, obesity rates are 10–27% in men and 10–38% in women, while in Poland, overweight or obesity affects more than 60 and 50% of the population, respectively. In contrast, abdominal obesity, which can occur in normal-weight individuals, affects 71% of women and 56% of men in Europe, while in Poland it affects 56% of men and 39% of women (8).

Carbohydrate metabolism disorders are one of the most important, and more difficult to treat, problems. The most common such disorder is type 2 diabetes, which has been declared an epidemic by the United Nations (UN) and the World Health Organization (WHO). It is noteworthy that it is the first and only disease whose cause is non-communicable and is attributed to an epidemic nature (9, 10). The disease, according to IDF statistics, affects 300 million people worldwide, however, as many as one-third do not receive treatment because they are unaware of the disease. Poland, like many other countries, is also struggling with this diabetes problem. A study showed that in the early 2000s, 5.6% of Poland’s population between the ages of 18 and 94 had diabetes, but the figure is still rising. Currently, 7% of men and 6% of women suffer from diabetes. Studies have recorded 2.17 million people with diabetes (1.22 million women and 0.96 million men). It is expected that by 2030 as much as 10% of the Polish population will have diabetes (10–12). Factors in the development of type 2 diabetes, are primarily improper eating habits, which include an inappropriate diet, poor in whole grains, vegetables, and fruits, while rich in saturated fatty acids, trans fats, and simple sugars. These factors are further reinforced by a lack of physical activity and chronic stress (10, 13–17).

The pre-diabetic state, in combination with the other elements of the metabolic syndrome, poses a serious threat to the patient’s health and even life. It is estimated that about 80–90% of patients struggling with diabetes are those who are simultaneously being treated for obesity, hypertension, as well as accompanying lipid disorders (12, 18).

Knowledge, attitudes, and practices related to nutrition play a key role in the prevention and treatment of obesity and metabolic diseases, such as type 2 diabetes and hypertension. Obesity, a global health issue, is often a result of poor dietary habits and lack of physical activity (14, 16). Research shows that nutritional education can significantly influence patient attitudes and behaviors, but knowledge alone does not always lead to improved health outcomes (15). Attitudes and motivation for change are equally important, though they may be hindered by external factors, such as access to healthy foods or social support. Regular adoption of healthy dietary habits can reduce the risk of complications related to obesity and diabetes, but maintaining these habits requires an integrated approach that includes education, behavioral support, and social reinforcement (15, 17).

The main objective of this study was to evaluate the dietary habits, nutritional knowledge, and nutritional status of patients hospitalized in the cardiology ward, taking into account the group of patients diagnosed with obesity or diabetes. It was hypothesized that hospitalized cardiac patients with obesity or diabetes show differences in eating habits and nutritional knowledge, which affect their nutritional status, compared to patients without these conditions.

## 2 Materials and methods

### 2.1 Study design

The study was conducted between January and June 2021. The site of the study was the Cardiology Department at the St. Barbara Regional Specialized Hospital No. 5 in Sosnowiec in Poland. Patients presented to the hospital with cardiovascular complaints (I00-I99, according to the International Classification of Diseases and Health Problems ICD-10). The study was conducted after obtaining permission from the department and approval from the Bioethics Committee of the Silesian Medical University in Katowice No. PCN/0022/KB/299/19/20 (approval date: 21.01.2020), patients invited to participate in the study were informed about the purpose of the study and expectations in accordance with the Declaration of Helsinki. Patients were included in the study after obtaining their verbal and written consent.

### 2.2 Study participants

The study group consisted of patients of the Cardiology Department at the St. Barbara Regional Specialized Hospital No. 5 in Sosnowiec. The group consisted of 301 patients, including 154 women (51.2%) and 147 men (48.8%), aged 29 to 87 years. Inclusion criteria for the study included (1) age over 18, (2) residence in the Silesian province, (3) diagnosis of cardiovascular disease, (4) stay in the Cardiology Department at the St. Barbara Regional Specialized Hospital No. 5 in Sosnowiec for at least 3 days, and (5) consent to the study. Exclusion criteria for the study included (1) the presence of mental disorders and other health conditions that prevented informed full and correct completion of the survey, and (2) incomplete completion of the survey.

### 2.3 Research tools

#### 2.3.1 Assessment of nutritional status

Nutritional status was assessed using the body mass index (BMI). The categorization was done according to the general findings of the WHO (World Health Organization): BMI <18.49 kg/m^2^—underweight, 18.5–24.99 kg/m^2^—normal weight, 25–29.99 kg/m^2^—overweight, 30–34.99 kg/m^2^ -obesity of the first degree, 35–39.99 kg/m^2^—obesity of II degree, >40 kg/m^2^—obesity of III degree (19).

#### 2.3.2 NRS 2002 scale

Patients’ nutritional status was also assessed by analyzing nutritional status and disease severity using the Nutritional Risk Score (NRS 2002) scale. According to Kondrup et al. (2, 3), the NRS-2002 nutritional risk score is based on an assessment of the following three items: nutritional status, disease severity, and age. Patients receive 0 to 3 points for nutritional status and 0 to 3 points for disease severity. A score of 1 is added to patients aged 70 and older. The total score ranges from 0 to 7, and if the total score is 3 or more, the patient is classified as at risk of malnutrition according to the NRS-2002. Nutritional status in the NRS-2002 is assessed by three individual components: BMI, recent weight loss (5% in the past 1, 2, or 3 months), and food intake in the previous week. Information on food intake during the week before hospital admission was determined by the dietitian through an interview with the patient. Questions focusing on food intake before hospitalization were compared with normal intake. In addition, food intake was divided into 0–25%, 25–50%, and 50–75% of normal requirements (20, 21).

#### 2.3.3 Assessment of dietary habits and nutritional knowledge

Individual face-to-face interviews were conducted by the researcher (nutritionist) to collect all relevant data from the study participants, including socio-demographic data (age, education level, place of residence) and lifestyle information. The study used a diagnostic survey method, within which a survey technique was applied. The author’s survey questionnaire consisted of a metric and two sections assessing dietary habits and knowledge.

The scope of the metric included questions on metric data such as gender, and age (determined by the year of birth and the year of the survey; environmental data: education; clinical data: diagnosed medical conditions, introduced diet). The author’s questionnaire examining the level of patients’ nutritional knowledge consisted of 19 closed-ended single-choice questions and two questions on self-assessment of their knowledge. The questionnaire examining patients’ eating habits included 9 closed-ended single-choice questions and a table of selected food products based on the FFQ-6 questionnaire (21). It assesses habitual consumption of food items over the past 12 months, divided into 6 levels of frequency. For each selected food item, respondents were able to indicate the frequency of their consumption by selecting one of the following options: (1) never, (2) once a month or less often, (3) several times a month, (4) several times a week, (5) daily or (6) several times a day.

A proprietary rating scale was used to assess the level of knowledge. Based on the questionnaire examining the level of nutritional knowledge and the answers given to questions 1–19, points were assigned: 0- incorrect answer, 1- correct answer. The total score for each patient and the percentage (%) against the maximum number of correct answers were then given. The level of knowledge was determined according to the following classification: Unsatisfactory 0–59%; Satisfactory 60–73%; Good 74–86%; Very good 87–100%.

A proprietary rating scale was used to assess the correctness of eating habits. Questions 1, 3, 6, 7, and 8 from Part I of the survey examining eating habits and all questions from Part II were evaluated. Based on the answers given in Part I, points were assigned: 0- incorrect answer, 1- correct answer. The answers to Part II, concerning the correctness of the frequency of consumption (never or seldom, once a month or less often, several times a month, several times a week, daily, several times a day) according to each food product, were assigned points: 0- answer may indicate incorrect eating habits, 1- correct answer. The total score for each patient and the percentage (W%) against the maximum number of correct answers were then given. The correctness of eating habits was determined according to the same classification as the level of nutritional knowledge.

#### 2.3.4 Morphology, biochemical blood tests

Blood samples from all participants were taken after a 12-h overnight fast. These tests included leukocyte (WBC), erythrocyte (RBC), platelet (PLT), hemoglobin (Hgb), and hematocrit (Hct) levels. Biochemical results included fasting blood glucose (FG), total cholesterol (TC), triglycerides (TG) LDL cholesterol (LDL-C), and HDL cholesterol (HDL-C), as well as sodium (Na) and potassium (K). Uric acid (UA), creatinine (Cr), troponin (Troponin T) alanine aminotransferase (ALT), aspartate aminotransferase (AST), and creatine kinase (CK-MB) levels were also performed.

#### 2.3.5 Blood pressure

Participants were in a sitting position for at least five minutes before blood pressure (BP) was assessed. A total of two trained nurses took two BP measurements for each participant (the same nurse took both measurements for a given participant) with a five-minute interval between measurements, according to Frese et al. (22). The mean value of each participant’s blood pressure calculated from the two measurements was used for the study.

### 2.4 Statistical analysis

Data were collected in MS Excel spreadsheet, MS Office 2013. Statistical analysis was performed using Statistica 2013, Stat Soft Poland. Measurable data were presented using measurable characteristics: mean (X) ± standard deviation (SD) median (M) and interquartile range (Rk). In the case of a symmetrical distribution, the mean was considered, while when the distribution was asymmetrical, the median value was interpreted. The Shapiro–Wilk test was used to assess the shape of the distribution. The analysis of differences in groups was conducted according to the number of groups and the shape of the distribution. In the case of two groups, the Student’s T-test or its non-parametric equivalent the Mann–Whitney U-test was used. For three or more groups, analysis of variance ANOVA or Kruskal-Wallis test was used, followed by multiple group analyses using post-hoc tests. Unmeasured data were presented as percentages. For analyses, significance tests of percentages within groups and χ2 tests of independence were used, taking into account corrections to the test (Yul’s test, Fischer’s test) depending on the expected numbers. In addition, correlation coefficients (Gamma, V Cramer) were provided to assess the strength of the relationship. In turn, to analyze the concordance of correct answers to questionnaire questions, Cochran’s Q test was performed. Wanting to assess the risk of metabolic syndrome, a logistic regression analysis was performed using the NRS 2002 level, age groups, and the results of morphological and biochemical blood tests. The level of statistical significance was set at *p* < 0.05.

## 3 Results

### 3.1 Characteristics of the study group

The study included 301 patients: 154 women (51.2%) and 147 men (48.8%), aged 29 to 87 years. They represented 5 age groups: under 50 (9.3%), 50–59 (12.6%), 60–69 (29.9%), 70–79 (36.9%) and over 80 (11.3%). Detailed information is presented in Table 1.

**Table 1:** Characteristics of the study group by gender and age.

		Gender			Age groups				
		Total	Women	Men	> 50	50–59	60–69	70–79	80 <
*N* (%)		301 (100)	154 (51.2)	147 (48.8)	28 (9.3)	38 (12.6)	90 (29.9)	111 (36.9)	34 (11.3)
Age [years] X ± SD		67 ± 11	68.7 ± 11.6	65.2 ± 10.1	43.5 ± 5.6	56 ± 2.1	64.7 ± 3.1	73.7 ± 2.5	82.7 ± 2
Body mass [kg] X ± SD		80.3 ± 14.7	75.2 ± 13	85.7 ± 14.5	82 ± 21	76.6 ± 16.6	84.5 ± 14.7	81.2 ± 11.4	69.5 ± 9.4
Height [cm] X ± SD		167.4 ± 9.6	161.3 ± 7	173.8 ± 7.4	175 ± 13.2	169.3 ± 5.1	168.4 ± 9.2	166 ± 9	161.2 ± 7.4
Education	Primary *N* (%)	15 (5)	11 (7.1)	4 (2.7)	3 (10.7)	0 (0)	6 (6.7)	4 (3.6)	2 (5.9)
	Secondary *N* (%)	155 (51.5)	69 (44.8)	86 (58.5)	7 (25)	15 (39.5)	62 (68.9)	61 (55)	10 (29.4)
	Vocational *N* (%)	72 (23.9)	40 (26)	32 (21.8)	6 (21.4)	17 (44.7)	16 (17.8)	20 (18)	13 (38.2)
	Higher *N* (%)	59 (19.6)	34 (22.1)	25 (17)	12 (42.9)	6 (15.8)	6 (6.7)	26 (23.4)	9 (26.5)

X = average; SD = standard deviation.

More than half of the cardiac patients studied were found to have obesity or diabetes (52.8%). There were no differences in the incidence of diabetes and obesity according to gender. However, there were differences in incidence according to age, those in the 70–79 age group were more likely to have diabetes and obesity (*p* < 0.01; *p* < 0.001). Details are shown in Table 2.

**Table 2:** Characteristics of the study group concerning the incidence of diabetes and obesity.

	Gender			*p*-value	Age groups					*p*-value
	Total *N* = 301 (100)	Women *N* = 154 (100)	Men *N* = 147 (100)		> 50 *N* = 28 (100)	50–59 *N* = 38 (100)	60–69 *N* = 90 (100)	70–79 *N* = 111 (100)	80 < *N* = 34 (100)	
Number of patients without diabetes or obesity *N* (%)	142 (47.2)	70 (45.5)	72 (49)	*p* = 0.42	14 (50)	16 (42.1)	51 (56.7)	42 (37.8)	19 (55.9)	*p* = 0.33
Number of patients with diabetes or obesity *N* (%)	159 (52.6)	84 (54.5)	75 (51)	*p* = 0.54	14 (50)	22 (57.9)	39 (43.3)	69 (62.2)	15 (44.1)	*p* = 0.07
Type 2 diabetes (E10-E14, ICD-10) *N* (%)	111 (36.8)	59 (38.3)	52 (35.4)	*p* = 0.59	3 (10.7)	15 (39.5)	28 (31.1)	53 (47.7)	12 (35.3)	*p* < 0.01*
Obesity (E65-E68, ICD-10) *N* (%)	103 (34.1)	59 (38.3)	44 (29.9)	*p* = 0.12	11 (39.3)	15 (39.5)	17 (18.9)	52 (46.8)	8 (23.5)	*p* < 0.001*

*p < 0.05.

### 3.2 BMI and NRS-2000

More than three-fourths of the study subjects (80%) showed above-normal body weight as interpreted by the BMI index. Obesity of the 1st and 2nd degree particularly affected women 27.9 and 14.9%, respectively. By age, grade I obesity was most common among those in the 70–79 age range (26.1%), while grade II obesity was most common among those aged 60–69 (24.4%). Details of the anthropometric data are shown in Table 3. There was no significant group variation by gender, age, or diagnosis of obesity or diabetes in the frequency of diagnosed nutritional level risk as measured by the NRS index (Table 4).

**Table 3:** Anthropometric data of patients in the study group.

Metric data		Total	Gender		Age groups				
			Women	Men	> 50	50–59	60–69	70–79	80 <
Total *N* (%)		301 (100)	154 (51.2)	147 (48.8)	28 (9.3)	38 (12.6)	90 (29.9)	111 (36.9)	34 (11.3)
Age [years]	X ± SD M (Rk)	67 ± 11 69 (13)	68.7 ± 11.6 70.5 (16)	65.2 ± 10.1 67 (14)	43.5 ± 5.6 45 (3)	56 ± 2.1 56 (3)	64.7 ± 3.1 64 (6)	73.7 ± 2.5 74 (4)	82.7 ± 2 83 (3)
Body weight [kg]		80.3 ± 14.7 80 (18)	75.2 ± 13 79 (19)	85.7 ± 14.5 84 (18)	82 ± 21 73.5(36.5)	76.6 ± 16.6 71 (25)	84.5 ± 14.7 83.5 (10)	81.2 ± 11.4 82 (16)	69.5 ± 9.4 72 (18)
Height [cm]		167.4 ± 9.6 168 (13)	161.3 ± 7 160 (10)	173.8 ± 7.4 172 (8)	175 ± 13.2 169 (29)	169.3 ± 5.1 168 (9)	168.4 ± 9.2 170 (11)	166 ± 9 166 (15)	161.2 ± 7.4 160 (11)
BMI [kg/m2]		28.7 ± 4.7 28.7 (5.6)	29 ± 4.9 29.4 (5.7)	28.3 ± 4.5 27.4 (4.1)	26.5 ± 4.9 26.3 (2.5)	26.7 ± 5.5 25.3 (7.8)	29.8 ± 4.9 29 (8)	29.5 ± 3.9 29.3 (4.4)	26.8 ± 3.3 26.7 (4.8)
BMI *N* (%)	Under-weight	6 (2)	6 (3.9)	0 (0)	3 (10.7)	0 (0)	0 (0)	3 (2.7)	0 (0)
	Normal weight	54 (17.9)	24 (15.6)	30 (20.4)	5 (17.9)	14 (36.8)	17 (18.9)	5 (4.5)	13 (38.2)
	Over-weight	143 (47.5)	58 (37.7)	85 (57.8)	17 (60.7)	17 (44.7)	34 (37.8)	62 (55.9)	13 (38.2)
	Obesity of the first degree	57 (18.9)	43 (27.9)	14 (9.5)	0 (0)	3 (7.9)	17 (18.9)	29 (26.1)	8 (23.5)
	Obesity of II degree	41 (13.6)	23 (14.9)	18 (12.2)	3 (10.7)	4 (10.5)	22 (24.4)	12 (10.8)	0 (0)

X = average; SD = standard deviation. M = median; Rk = range.

**Table 4:** Analysis of nutritional status risk using the NRS-2002 index.

Predictors		Total *N* = 301 (100)	NRS 2002					*p*-value
			NRS = 0 *N* = 41	NRS = 1 *N* = 122	NRS = 2 *N* = 102	NRS = 3 *N* = 21	NRS = 4 *N* = 15	
Gender	Women *N* = 154	154 (51.2)	22 (14.3)	66 (42.9)	48 (31.2)	10 (6.5)	8 (5.2)	*p* = 0.85370
	Men *N* = 147	147 (48.8)	19 (12.9)	56 (38.1)	54 (36.7)	11 (7.5)	7 (4.8)	
Age groups	> 50 *N* = 28	28 (9.3)	4 (14.3)	9 (32.1)	13 (46.4)	2 (7.1)	0 (0)	*p* = 0.54238
	50–59 *N* = 38	38 (12.6)	4 (10.5)	21 (55.3)	9 (23.7)	2 (5.3)	2 (5.3)	
	60–69 *N* = 90	90 (29.9)	12 (13.3)	40 (44.4)	30 (33.3)	4 (4.4)	4 (4.4)	
	70–79 *N* = 111	111 (36.9)	14 (12.6)	39 (35.1)	42 (37.8)	10 (9)	6 (5.4)	
	80 < *N* = 34	34 (11.3)	7 (20.6)	13 (38.2)	8 (23.5)	3 (8.8)	3 (8.8)	
Diabetes or obesity	No *N* = 142	142 (47.2)	14 (9.9)	63 (44.4)	47 (33.1)	12 (8.5)	6 (4.2)	*p* = 0.28457
	Yes *N* = 159	159 (52.8)	27 (17)	59 (37.1)	55 (34.6)	9 (5.7)	9 (5.7)	

### 3.3 Nutrition knowledge

The results of the patient’s nutritional knowledge survey were mixed. Patients with diabetes and/or obesity had better knowledge of the source of dietary protein (84.3% vs. 66.9%), the function and source of dietary fiber (52.8% vs. 37.3% and 52.2% vs. 48.6%, respectively), the function of iron (63.5% vs. 42.3%), and the source of vitamin C (71.7% vs. 58.5%). Patients who were not diagnosed with these entities did better on questions about dietary sources of calcium (81.8% vs. 57.1%). Among all respondents, respondents gave the most correct answers in questions about the source of protein (76.1%), the source of calcium (69.8%), and the source of vitamin C (65.4%). The exact results are shown in Table 5.

**Table 5:** Respondents’ nutritional knowledge.

Nutritional knowledge		Total *N* = 301 (100)	Diabetes and/or obesity		*p*-value
Question	Correct answer		No *N* = 142 (100)	Yes *N* = 159 (100)	
How many meals should be eaten in a day?	4–5 meals	182 (60.5)	80 (56.3)	102 (64.2)	*p* = 0.53774
Intervals between meals should be no more than:	3–4 h	211 (70.1)	95 (66.9)	116 (73)	*p* = 0.33045
The most recommended heat treatment of food is:	Cooking	190 (63.1)	93 (65.5)	97 (61)	*p* = 0.06109
The primary function of protein in the human body is:	Building function	96 (31.9)	50 (35.2)	46 (28.9)	*p* = 0.00519*
The best sources of protein in the diet are:	Meat, fish, eggs	229 (76.1)	95 (66.9)	134 (84.3)	*p* = 0.00678*
The best sources of fat in the diet are:	Vegetable oils and marine fish	244 (81.1)	117 (82.4)	127 (79.9)	*p* = 0.08984
Dietary fiber is responsible for:	Regulation of intestinal motility	137 (45.5)	53 (37.3)	84 (52.8)	*p* < 0.0001
The best sources of dietary fiber are:	Cereal products, vegetables	152 (50.5)	69 (48.6)	83 (52.2)	*p* = 0.00318*
The best sources of sodium are:	Meat and meat products	61 (20.3)	33 (23.2)	28 (17.6)	*p* = 0.61551
The best sources of potassium are:	Legumes	194 (64.5)	91 (64.1)	103 (64.8)	*p* = 0.59756
What can cause an excess of potassium in the diet?	Heart rhythm disorders	171 (56.8)	85 (59.9)	86 (54.1)	*p* = 0.02551*
The best sources of phosphorus are:	Cottage cheese and egg white	36 (12)	20 (14.1)	16 (10.1)	*p* = 0.13487
A proper supply of calcium prevents:	Osteoporosis	165 (54.8)	85 (59.9)	80 (50.3)	*p* = 0.56579
The best sources of calcium in the diet are:	Milk and dairy products	210 (69.8)	126 (81.8)	84 (57.1)	*p* < 0.0001
Iron is the element responsible for:	Oxygen transport in the body	161 (53.5)	60 (42.3)	101 (63.5)	*p* = 0.00335*
The best dietary sources of iron are:	Meat products	107 (35.5)	55 (38.7)	52 (32.7)	*p* = 0.25639
Fat-soluble vitamins include:	A, D, E, K	82 (27.2)	42 (29.6)	40 (25.2)	*p* = 0.27905
The best sources of vitamin D are:	Sunlight	185 (61.5)	87 (61.3)	98 (61.6)	*p* = 0.21427
The best sources of vitamin C are:	Fruits and vegetables	197 (65.4)	83 (58.5)	114 (71.7)	*p* = 0.00194*

*=*p* < 0.05.

**Figure 1:**
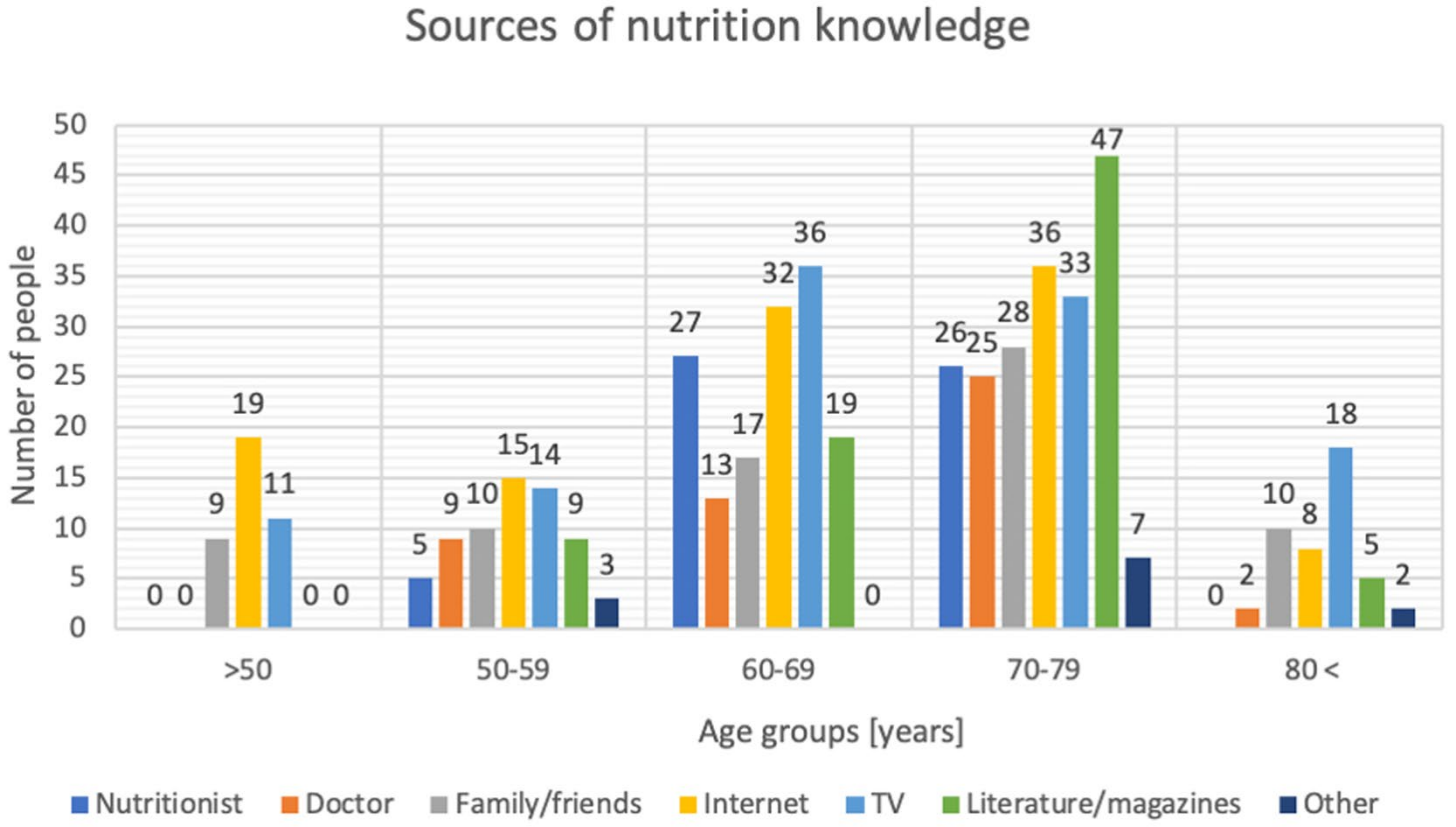
Respondents’ sources of nutritional knowledge by age (*n*=301).

When asked about sources of nutritional knowledge, men were more likely to indicate that a dietician was the source of their knowledge relative to women’s indications (*p* = 0.005), while women were more likely to indicate literature and magazines (*p* = 0.001). Analyzing the sources of knowledge in the age groups studied showed that those aged 60–69 were significantly more likely to indicate that a nutritionist was their source of knowledge (*p* = 0.0001). In contrast, those aged 70–79 were significantly more likely to indicate a doctor. The Internet was the primary source of information for those under 50, with 67.9% indicating this response (*p* = 0.01). Detailed information is shown in Figure 1.

### 3.4 Eating habits

Respondents were mostly characterized by incorrect eating habits. Only 26.2% of respondents always eat a second breakfast, and 44.3% of them choose fruit for this meal. A disturbing result is that only 9.3% of the subjects, including 15.7% with obesity and/or diabetes and only 2.1% without these diseases, chose water as a beverage for their meal. It is noteworthy, however, that almost ¾ of patients, regardless of the diagnosed disease entity, are wary of overeating at night (Table 6). The study of dietary habits in terms of frequency of intake of selected nutrients was divided into food groups such as cereal products, dairy products and eggs, meat products and fish, vegetables and grains, fruits, fats, beverages, and sweets. Detailed results including the above groups are shown in Table 7.

**Table 6:** Eating habits of respondents.

Eating habits		Total *N* = 301 (100)	Obesity and/or diabetes		*p*-value
			No *N* = 142 (100)	Yes *N* = 159 (100)	
Question	Correct answer				
Does he eat I breakfast in the morning?	Yes, always	251 (83.4)	119 (83.8)	132 (83)	*p* = 0.08551
What does he eat most often for I breakfast?	A sandwich with cheese or cold cuts and vegetables	97 (32.2)	31 (21.8)	66 (41.5)	*p* = 0.00027
Does he eat the 2nd breakfast?	Yes, always	79 (26.2)	46 (32.4)	33 (20.8)	*p* = 0.02846*
What does he consume most often for II breakfast?	Fruit	98 (44.3)	37 (35.6)	61 (52.1)	*p* = 0.00023*
What does he drink most often with his meal?	Non-carbonated mineral water	28 (9.3)	3 (2.1)	25 (15.7)	*p* < 0.0001
How many meals does he eat most often during the day?	4–5 meals	132 (43.9)	67 (47.2)	65 (40.9)	*p* = 0.12395
Does he/she happen to overeat between meals?	No, never	46 (15.3)	18 (12.7)	28 (17.6)	*p* = 0.19110
Does he happen to overeat at night?	No, never	218 (72.4)	107 (75.4)	111 (69.8)	*p* = 0.00079*
Does he consider his nutrition to be healthy?	No	43 (14.3)	15 (10.6)	28 (17.6)	*p* = 0.19904

*=*p* < 0.05.

**Table 7:** Frequency of consumption of selected products by respondents.

Eating habits (frequency of consumption)		Total, *N* = 301 (100)	Obesity and/or diabetes		*p*-value
			No *N* = 142 (100)	Yes *N* = 159 (100)	
Cereal products					
Wholemeal bread	Several times a week	79 (26.2)	33 (23.2)	46 (28.9)	*p* = 0.09367
	Daily	80 (26.6)	43 (30.3)	37 (23.3)	
	Several times a day	10 (3.3)	1 (0.7)	9 (5.7)	
Refined bread	Never or seldom	32 (10.6)	14 (9.9)	18 (11.3)	*p* = 0.09702
	Once a month or less often	32 (10.6)	15 (10.6)	17 (10.7)	
	Several times a month	101 (33.6)	43 (30.3)	58 (36.5)	
Coarse-grain groats	Several times a month	173 (57.5)	78 (54.9)	95 (59.7)	*p* = 0.44252
	Several times a week	51 (16.9)	24 (16.9)	27 (17)	
	Daily	2 (0.7)	2 (1.4)	0 (0)	
Fine-grained groats	Never or seldom	15 (5)	7 (4.9)	8 (5)	*p* = 0.11318
	Once a month or less often	48 (15.9)	16 (11.3)	32 (20.1)	
	Several times a month	173 (57.5)	90 (63.4)	83 (52.2)	
Dairy products and eggs					
Milk and natural dairy drinks	Several times a week	112 (37.2)	52 (36.6)	60 (37.7)	*p* = 0.21595
	Daily	26 (8.6)	11 (7.7)	15 (9.4)	
	Several times a day	3 (1)	1 (0.7)	2(1.3)	
Sweetened dairy beverages	Never or seldom	79 (26.2)	39 (27.5)	40 (25.2)	*p* = 0.11263
	Once a month or less often	84 (27.9)	35 (24.6)	49 (30.8)	
	Several times a month	100 (33.2)	54 (38)	46 (28.9)	
Cheeses	Never or seldom	13 (4.3)	5 (3.5)	8 (5)	*p* = 0.63794
	Once a month or less often	72 (23.9)	29 (20.4)	43 (27)	
	Several times a month	115 (38.2)	60 (42.3)	55 (34.6)	
Eggs and egg dishes	Several times a month	166 (55.1)	84 (59.2)	82 (51.6)	*p* = 0.00861*
	Several times a week	71 (23.6)	30 (21.1)	41 (25.8)	
Meat products and fish					
Sausages	Never or seldom	22 (7.3)	11 (7.7)	11 (6.9)	*p* = 0.00113*
	Once a month or less often	33 (11)	12 (8.5)	21 (13.2)	
Premium cured meats	Once a month or less often	7 (2.3)	6 (4.2)	1 (0.6)	*p* = 0.16647
	Several times a month	135 (44.9)	61 (43)	74 (46.5)	
	Several times a week	124 (41.2)	59 (41.5)	65 (40.9)	
Sausage products and offal meat	Never or seldom	36 (12)	19 (13.4)	17 (10.7)	*p* = 0.00542*
	Once a month or less often	92 (30.6)	37 (26.1)	55 (34.6)	
	Several times a month	121 (40.2)	67 (47.2)	54 (34)	
Red meat	Never or seldom	23 (7.6)	13 (9.2)	10 (6.3)	*p* = 0.13540
	Once a month or less often	71 (23.6)	32 (22.5)	39 (24.5)	
	Several times a month	132 (43.9)	70 (49.3)	62 (39)	
Poultry and rabbit meat	Several times a month	142 (47.2)	64 (45.1)	78 (49.1)	*p* = 0.07280
	Several times a week	112 (37.2)	49 (34.5)	63 (39.6)	
Lean fish	Several times a month	157 (52.2)	79 (55.6)	78 (49.1)	*p* = 0.06598
	Several times a week	50 (16.6)	28 (19.7)	22 (13.8)	
Fatty fish	Several times a month	125 (41.5)	66 (46.5)	59 (37.1)	*p* = 0.47977
	Several times a week	59 (19.6)	27 (19)	32 (20.1)	
Vegetables and grains					
Cruciferous vegetables	Several times a week	110 (36.5)	49 (34.5)	61 (38.4)	*p* = 0.37407
	Daily	20 (6.6)	13 (9.2)	7 (4.4)	
Yellow-orange vegetables	Several times a week	120 (39.9)	54 (38)	66 (41.5)	*p* = 0.13346
	Daily	32 (10.6)	17 (12)	15 (9.4)	
	Several times a day	3 (1)	1 (0.7)	2 (1.3)	
Green leafy vegetables	Several times a week	112 (37.2)	54 (38)	58 (36.5)	*p* = 0.26660
	Daily	16 (5.3)	4 (2.8)	12 (7.5)	
	Several times a day	3 (1)	1 (0.7)	2 (1.3)	
Tomatoes	Several times a week	114 (37.9)	45 (31.7)	69 (43.4)	*p* = 0.30466
	Daily	104 (34.6)	56 (39.4)	48 (30.2)	
	Several times a day	16 (5.3)	9 (6.3)	7 (4.4)	
Cucumbers	Several times a week	160 (53.2)	72 (50.7)	88 (55.3)	*p* = 0.13872
	Daily	34 (11.3)	23 (16.2)	11 (6.9)	
	Several times a day	8 (2.7)	3 (2.1)	5 (3.1)	
Root vegetables	Several times a week	126 (41.9)	58 (40.8)	68 (42.8)	*p* = 0.38441
	Daily	26 (8.6)	15 (10.6)	11 (6.9)	
	Several times a day	5 (1.7)	3 (2.1)	2 (1.3)	
Dry pulses	Once a month or less often	127 (42.5)	64 (45.7)	63 (39.6)	*p* = 0.23453
	Several times a month	87 (29.1)	44 (31.4)	43 (27)	
Potatoes	Several times a week	79 (26.2)	50 (35.2)	29 (18.2)	*p* = 0.00303*
	Daily	78 (25.9)	36 (25.4)	42 (26.4)	
Nuts	Several times a week	57 (18.9)	31 (21.8)	26 (16.4)	*p* = 0.75751
	Daily	6 (2)	3 (2.1)	3 (1.9)	
	Several times a day	6 (2)	2 (1.4)	4 (2.5)	
Grains	Several times a month	77 (25.6)	33 (23.2)	44 (27.7)	*p* = 0.23060
	Several times a week	37 (12.3)	17 (12)	20 (12.6)	
	Daily	2 (0.7)	2 (1.4)	0 (0)	
Fruits					
Stone fruits	Several times a week	101 (33.6)	46 (32.4)	55 (34.6)	*p* = 0.85788
	Daily	28 (9.3)	13 (9.2)	15 (9.4)	
Kiwis and citrus	Several times a week	79 (26.2)	39 (27.5)	40 (25.2)	*p* = 0.46113
	Daily	26 (8.6)	12 (8.5)	14 (8.8)	
Tropical fruits	Several times a week	36 (12)	21 (14.8)	15 (9.4)	*p* = 0.40153
	Daily	6 (2)	4 (2.8)	2 (1.3)	
Berry fruits	Several times a week	97 (32.2)	41 (28.9)	56 (35.2)	*p* = 0.62881
	Daily	9 (3)	3 (2.1)	6 (3.8)	
Bananas	Several times a week	78 (25.9)	12 (8.5)	15 (9.4)	*p* = 0.10698
	Daily	27 (9)	0 (0)	4 (2.5)	
Apples and pears	Several times a week	86 (28.6)	52 (36.6)	34 (21.4)	*p* = 0.00410*
	Daily	55 (18.3)	29 (20.4)	26 (16.4)	
Dried fruits	Several times a week	39 (13)	20 (14.1)	19 (11.9)	*p* = 0.38912
	Daily	8 (2.7)	5 (3.5)	3 (1.9)	
Sweet fruit preserves and candied fruits	Never or seldom	51 (16.9)	24 (16.9)	27 (17)	*p* = 0.75277
	Once a month or less often	91 (30.2)	38 (26.8)	53 (33.3)	
	Several times a month	94 (31.2)	48 (33.8)	46 (28.9)	
Fats					
Oil	Several times a week	100 (33.2)	54 (38)	46 (28.9)	*p* = 0.00158
	Every day	90 (29.9)	37 (26.1)	53 (33.3)	
Butter	Never or seldom	10 (3.3)	2 (1.4)	8 (5)	*p* = 0.04389*
	Once a month or less often	16 (5.3)	8 (5.6)	8 (5)	
	Several times a month	43 (14.3)	20 (14.1)	23 (14.5)	
Cream	Never or seldom	20 (6.6)	11 (7.7)	9 (5.7)	*p* = 0.78537
	Once a month or less often	47 (15.6)	21 (14.8)	26 (16.4)	
	Several times a month	76 (25.2)	36 (25.4)	40 (25.2)	
Animal fats	Never or seldom	86 (28.6)	44 (31)	42 (26.4)	*p* = 0.00169*
	Once a month or less often	65 (21.6)	23 (16.2)	42 (26.4)	
Mayonnaise and dressings	Never or seldom	63 (20.9)	28 (19.7)	35 (22)	*p* = 0.12844
	Once a month or less often	80 (26.6)	42 (29.6)	38 (23.9)	
	Several times a month	84 (27.9)	35 (24.6)	49 (30.8)	
Drinks					
Fruit juices and nectars	Several times a month	103 (34.2)	54 (38)	49 (30.8)	*p* = 0.05063
	Several times a week	56 (18.6)	24 (16.9)	32 (20.1)	
Vegetable and fruit and vegetable juices	Several times a week	71 (23.6)	38 (26.8)	33 (20.8)	*p* = 0.16588
	Daily	18 (6)	11 (7.7)	7 (4.4)	
Hot drinks	Never or seldom	10 (3.3)	6 (4.2)	4 (2.5)	*p* = 0.72445
	Once a month or less often	7 (2.3)	4 (2.8)	3 (1.9)	
	Several times a month	21 (7)	11 (7.7)	10 (6.3)	
Energy drinks	Never or seldom	183 (60.8)	77 (54.2)	106 (66.7)	*p* = 0.11459
Sweetened beverages	Never or seldom	148 (49.2)	71 (50)	77 (48.4)	*p* = 0.61921
Beer	Never or seldom	113 (37.5)	46 (32.4)	67 (42.1)	*p* = 0.12743
	Once a month or less often	60 (19.9)	31 (21.8)	29 (18.2)	
Vodka and spirits	Never or seldom	122 (40.5)	48 (33.8)	74 (46.5)	*p* = 0.07398
Sweets					
Sugar	Never or seldom	83 (27.6)	32 (22.5)	51 (32.1)	*p* = 0.13781
Honey	Several times a month	55 (18.3)	29 (20.4)	26 (16.4)	*p* = 0.00027*
	Several times a week	87 (28.9)	43 (30.3)	44 (27.7)	
	Every day	25 (8.3)	19 (13.4)	6 (3.8)	
Chocolate and chocolate candies	Never or seldom	32 (10.6)	13 (9.2)	19 (11.9)	*p* = 0.06758
Candy without chocolate	Never or seldom	52 (17.3)	20 (14.1)	32 (20.1)	*p* = 0.06419
Cookies and cakes	Never or seldom	14 (4.7)	4 (2.8)	10 (6.3)	*p* = 0.23284
Ice cream and pudding	Never or seldom	39 (13)	17 (12)	22 (13.8)	*p* = 0.74467
Salty snacks	Never or seldom	120 (39.9)	54 (38)	66 (41.5)	*p* = 0.21015

*=*p* < 0.05.

### 3.5 Biochemistry and blood pressure

There was not much variation in the levels of blood biochemical parameters among the groups classified by BMI. The presence of obesity and/or diabetes generally did not affect the variation in levels of blood biochemical parameters. The results were not statistically significant. Detailed information is presented in Table 8.

**Table 8:** Analysis of blood pressure and blood biochemistry results.

Biochemical parameters of blood	Total *N* = 301 X ± SD	Diabetes and/or obesity		*p*-value
		No *N* = 142 X ± SD	Yes *N* = 159 X ± SD	
SBP [mmHg]	140.8 ± 20 14	141.5 ± 19.4 145	140.3 ± 20.5 140	*p* = 0.467
DBP [mmHg]	79.7 ± 12 80	81.2 ± 12.9 80	78.4 ± 11 80	*p* = 0.034
WBC [tys./μl]	8.3 ± 3.1 7.8	8.3 ± 3.2 7.7	8.4 ± 3 7.9	*p* = 0.369
RBC [mln/μl]	4.6 ± 0.8 4.6	4.7 ± 0.9 4.6	4.6 ± 0.6 4.6	*p* = 0.656
Hgb [g/dl]	17 ± 18.9 13.8	14.1 ± 5.3 13.7	19.5 ± 25.3 14.2	*p* = 0.043*
Hct [%]	40,6 ± 4.3 41	40.4 ± 4.1 40.7	40.9 ± 4.5 41.2	*p* = 0.245
PLT [tys./mm^3^]	228.2 ± 73 220	224.9 ± 72.1 217	231.1 ± 73.8 221	*p* = 0.595
Na [mmol/l]	138.3 ± 3.7 138	138.6 ± 4.4 138	138 ± 2.9 138	*p* = 0.204
K [mmol/l]	4.3 ± 0.5 4.2	4.2 ± 0.5 4.2	4.3 ± 0.5 4.2	*p* = 0.656
AST [IU/l]	30 ± 13.1 26	31.1 ± 14 27	29 ± 12.2 25	*p* = 0.115
ALT [IU/l]	26.4 ± 14 23	27 ± 14 23	25.8 ± 13.9 21	*p* = 0.319
CK-MB [ng/ml]	16.9 ± 10.3 14	16.9 ± 10.2 14	16.8 ± 10.5 15	*p* = 0.970
Troponin T [μg/l]	1415.1 ± 6266.3 9.6	2036.2 ± 7962.8 11.2	860.4 ± 4161.4 9.4	*p* = 0.278
TC [mg/dl]	186.4 ± 52 179	184.3 ± 44.6 182	188.2 ± 57.9 179	*p* = 0.872
HDL-C [mg/dl]	56.5 ± 18.3 54	56.9 ± 17.5 55	56.2 ± 19 52	*p* = 0.383
LDL-C [mg/dl]	115.9 ± 38.1 107	114.3 ± 34.2 107	117.4 ± 41.4 109	*p* = 0.871
TG [mg/dl]	143.8 ± 154.8 116	136.1 ± 99.7 115	150.6 ± 191.1 116	*p* = 0.705
Cr [mg/dl]	1.2 ± 1.2 1	1.2 ± 1.1 1	1.3 ± 1.3 1	*p* = 0.786
FC [mg/dl]	122.8 ± 49.6 109	121.4 ± 45.5 107.5	124 ± 53.1 110	*p* = 0.395
CRP [mg/l]	14.6 ± 37.8 4.5	16.3 ± 42.5 4.7	13.1 ± 33.1 3.6	*p* = 0.464
UA [mg/dl]	6.2 ± 2 5.9	6.3 ± 2.1 6	6.1 ± 1.9 5.9	*p* = 0.449
D-Dimery [ng FEU/ml]	2.5 ± 3.7 0.8	3 ± 4.5 1.1	1.9 ± 2 0.8	*p* = 0.764

X = average; SD = standard deviation. * = *p* < 0.05.

## 4 Discussion

The purpose of this study was to evaluate and analyze in detail the knowledge, behavior, and eating habits of patients hospitalized in the cardiology department with diagnosed diabetes or obesity, taking into account the results of morphological and biochemical tests and their nutritional status. The study included 301 patients hospitalized in the Cardiology Department of a hospital in the Silesian province. A survey technique based on proprietary questionnaires and available methods of assessing nutritional status was used. The study was conducted from January to June 2021 with the approval of the hospital director.

The hypothesis was that people with diabetes or obesity would show differences in diet and nutrition knowledge, which would affect their nutritional status compared to those without these conditions. The results of the study showed that more than 65% of overweight or obese respondents scored either sufficient or good on the nutrition knowledge questionnaire. In comparison, only 24.1% of those with a normal BMI scored other than inadequate. These results suggest that overweight individuals have greater nutritional awareness, although this does not always translate into healthy eating habits.

Respondents declared eating mainly 4–5 meals a day, and most of them, regardless of the type of health ailment, had breakfast every day, choosing most often a cheese or ham sandwich and tea. The highest daily consumption of wholemeal baked goods was declared by overweight and obese respondents. Similar results were obtained by Stefanska et al. (23)-in their study, about 32% of obese people declared daily consumption of wholemeal products. The study by Pielak et al. (24) showed that both rye bread and wheat bread are consumed with the same frequency, while diet, health-promoting, and crisp bread are far less popular among the surveyed consumers. Comparing the data concerning obesity and diabetes, it can be concluded that the results in all groups are similar.

Analysis of the weekly consumption of dairy products did not show particularly high consumption of these products in the diet of the subjects—the results oscillated around 37%. The relationship between the consumption of dairy products and eggs and the occurrence of obesity or diabetes could not be established. A comparison of data on the consumption of different types of meat showed that the respondents consumed lean fish, poultry, and red meats to a similar extent while avoiding organ meats and sausage products. This was also confirmed by a study conducted in Wroclaw in 2010 (25). The results showed that for 54% of women and 36% of men with hypertension, the presence of fat in the food items in question was of great importance when purchasing meat and meat products.

Regardless of the diagnosis of diabetes or obesity, patients were most likely to choose cucumber, root, and leafy vegetables. In comparison, the results of a study conducted during training at the Biaton Health Academy by Wlodarek and Glabska showed that patients with diabetes t. 2 were most likely to eat tomatoes, carrots, and cabbage (26). Regardless of BMI and prevalent diseases, the least frequently chosen products daily are nuts, pulses, and grains. The same results were presented in a study by Drywiew and Kuć (27), which proved the low consumption of legumes by elderly people living in rural areas.

Analyzing the results, it can be concluded that apples are the most consumed fruits daily. This is understandable, as they are available all year round, relatively cheap, and firmly rooted in Polish culinary tradition. Dried and candied fruits were chosen on average once a month or less often. Daily consumption of butter was declared by all respondents, regardless of the diagnosed disease entity. Consumption of mayonnaise and dressings was limited to a few times a month, which is a positive surprise considering the high energy value of these products. Similar results were obtained by Pilska (28), whose study of a representative group of Poles showed that butter was the most commonly consumed type of fat, followed by canola oil, margarine, olive oil, and sunflower oil.

All respondents unanimously avoided the consumption of sweetened and energy drinks—more than 60% never or seldom included them in their diet. This fact is a positive but unsurprising development. The respondents are mainly people aged 50 to 80 and older, who did not use these types of drinks during the acquisition of their eating habits (childhood and adolescence), so it was likely that the percentage of consumption of these products would be low. During the daily period, hot beverages are the most popular, with the majority of respondents declaring that they consume them daily or drink them even several times a day. Different results are presented in the study by Witanowska et al. (29), according to which the respondents are most likely to drink mineral water (83%), followed by fruit juices and sweet drinks. However, it is important to note the negligible consumption of energy drinks, both in this analysis and in our study.

Respondents also avoided the consumption of alcoholic beverages, with the highest consumption of wine, drinks, vodka, and spirits declared as “never” or “seldom” and “once a month” or less often. Analyzing the consumption of sweets, all respondents most often reached for chocolates, chocolate products, cookies, cakes, ice cream, puddings, and non-chocolate candies several times a month, with the percentage of consumption of each category of items being similar in all groups.

Summarizing the respondents’ knowledge and eating habits, it can be concluded that they vary depending on the topic addressed, but are generally incorrect about the diagnosed disease entity. Similar results were obtained in a study by Bieniek-Walenda et al. (30), who assessed the level of nutritional knowledge of patients after acute coronary syndrome hospitalized in a cardiology department. A good level of nutritional knowledge concerned only 6.9% of the subjects.

Analyzing dietary habits and the effect of diet on glycemic variability in patients with type 1 diabetes, a study by Koperska et al. (31) observed that, except for too little consumption of vegetables and too much consumption of fatty acids, the diet of respondents was satisfactory and following recommendations. Diet significantly influenced the variability of glycemic results, and properly planned nutrition was conducive to achieving the intended therapeutic goals. A study by Izycka et al. (32) on the diet of another group of patients, i.e., patients diagnosed with non-alcoholic steatohepatitis, showed that the vast majority of respondents, as in the author’s study, were eating incorrectly. Mistakes consisted mainly of consuming too many products rich in saturated fatty acids and simple sugars.

The results of assessing the dietary habits of patients with cardiovascular disease in the study by Mikulska et al. (33) were similar to those of the authors. The subjects also consumed excessive amounts of foods rich in fat and simple sugars, and insufficient amounts of foods containing dietary fiber, vegetables, legumes, nuts and fish. Similar to our results, the study also reported insufficient water intake. In conclusion, regardless of the diagnosed disease, the level of knowledge and eating habits of the public is inadequate, and it is necessary to introduce broad-based nutrition education.

Tests on the levels of biochemical parameters and patients’ blood counts, as well as the respondents’ blood pressure and their NRS, did not correlate with the occurrence of diabetes and/or obesity. Different results were obtained by Lewandowski et al. (34), who compared blood morphology studies with selected biochemistry results in elderly patients. This study showed that the onset of inflammation and associated changes in biochemistry affect the morphology of responders.

One of the main strengths of the study is the large number of respondents, which increases the representativeness of the results and allows for more reliable conclusions about the dietary habits and nutritional status of patients with cardiovascular disease. The use of the FFQ questionnaire provides accurate results on the frequency of product consumption, which allows for a detailed analysis of eating habits. In addition, a detailed questionnaire assessing the level of patients’ nutritional knowledge makes it possible to evaluate their awareness of diet and its impact on health. The survey took into account both clinical data (e.g., blood pressure, morphological and biochemical blood test results) and information on dietary habits, which allows a broad assessment of the impact of diet on patients’ health. It should be mentioned that the study was conducted in a hospital setting allowed direct assessment of patients’ health status and provided greater control over the quality of the data collected.

However, the study also has some weaknesses. Limiting the survey to a single institution may limit the ability to generalize the results to a broader population of patients with cardiovascular disease. There is also a risk of potential respondent error, as with surveys there is always the possibility that respondents may not answer honestly or accurately, which may affect the reliability of the results. In addition, the measurement of dietary habits and nutritional status was conducted only once, which does not allow for analysis of changes over time.

## 5 Conclusion

Analysis of the results obtained allows us to propose conclusions that the body weight and BMI value of the majority of respondents indicated overweight or obesity, and these results varied by gender, age, and the presence of diseases. There was no increased risk of malnutrition and no variation in the prevalence of malnutrition levels using the NRS 2002 scale. In addition, the respondents’ level of knowledge was inadequate and varied. Several abnormalities in dietary behavior and habits were found, in particular: a low level of consumption of vegetables and fruits, pulses, nuts and grains, whole grain cereal products, fish and milk, and dairy products, as well as a markedly insufficient intake of water. On the positive side, there was a low intake of salty snacks, sweetened energy drinks, and alcohol. There were no differences in the respondents’ eating behavior and habits in the context of the diagnosed disease entity. In addition, the vast majority of respondents had a diagnosed metabolic disease, co-morbidities were also common, and analysis of biochemical and blood count results showed some abnormalities, but these results did not vary by gender, age, BMI, and NRS scale.

The study results clearly indicate significant clinical and public health implications. Clinically, the inappropriate body weight of the majority of patients with cardiovascular diseases significantly increases the risk of cardiovascular complications. Although patients with diabetes and obesity possess better nutritional knowledge, this does not always translate into healthy dietary habits, highlighting the need for education that effectively changes behaviors. From a public health perspective, these findings emphasize the necessity of spreading nutritional education throughout society, especially among high-risk groups, and implementing educational programs and policies that promote healthy eating. Early dietary intervention based on solid knowledge could significantly reduce the risk of developing chronic diseases, thereby improving the overall health of the population.

## Data Availability

The raw data supporting the conclusions of this article will be made available by the authors, without undue reservation.
